# Development and validation of the Maugeri Sleep Quality and Distress Inventory (MaSQuDI-17)

**DOI:** 10.1371/journal.pone.0180743

**Published:** 2017-07-10

**Authors:** Elisa Morrone, Cinzia Sguazzin, Giorgio Bertolotti, Andrea Giordano, Alberto Braghiroli, Gian Luigi Balestroni, Raffaele Manni, Luigi Ferini Strambi, Vincenza Castronovo, Marco Zucconi, Fabrizio De Carli, Eleonora Pinna, Marcella Ottonello, Ines Giorgi, Michele Terzaghi, Sara Marelli, Francesco Fanfulla

**Affiliations:** 1 Sleep Medicine Unit, Istituti Clinici Scientifici Maugeri, Scientific Istitute of Pavia IRCCS, Pavia, Italy; 2 Psychology Unit, Istituti Clinici Scientifici Maugeri, Scientific Istitute of Pavia IRCCS, Pavia Italy; 3 Psychology Unit, ICS Maugeri, Istituti Clinici Scientifici Maugeri, Scientific Istitute of Tradate IRCCS, Tradate, Italy; 4 Unit of Bioengineering, Istituti Clinici Scientifici Maugeri, Scientific Istitute of Veruno IRCCS, Veruno, Italy; 5 Division of Respiratory Disease, Istituti Clinici Scientifici Maugeri, Scientific Istitute of Veruno IRCCS, Veruno, Italy; 6 Psychology Unit, Istituti Clinici Scientifici Maugeri, Scientific Istitute of Veruno IRCCS, Veruno, Italy; 7 Unit of Sleep Medicine and Epilepsy, C. Mondino National Neurological Institute, Pavia, Italy; 8 Department of Clinical Neurosciences, San Raffaele Scientific Institute, Sleep Disorders Center, Università Vita-Salute San Raffaele, Milano, Italy; 9 Institute of Bioimaging and Molecular Physiology, National Research Council (CNR) Genova, Italy; 10 Psychology Unit, Istituti Clinici Scientifici Maugeri, Scientific Istitute of Genova Nervi, Genova,Italy; 11 Psychology Unit, Istituti Clinici Scientifici Maugeri, Scientific Istitute of Pavia IRCCS, Pavia, Italy; Universita Cattolica del Sacro Cuore Sede di Roma, ITALY

## Abstract

**Objectives:**

The aim of this study was to develop and validate a questionnaire designed to measure the impact of sleep impairment on emotional distress in patients with various sleep disorders.

**Methods:**

Five experts created an item data-bank pertaining to sleep-related psychological symptoms and somatic perceptions. Fifty patients in two focus groups examined each item for: a) word clarity (indicating any ambiguity of interpretation) and b) appropriateness for the target population. This process permitted to identify 36 appropriate items. Classical Test Theory and Rasch Analysis were used to further refine the questionnaire, yielding the final 17-item set. Concurrent validation of the new scale was tested with the Pittsburgh Sleep Quality Index, Epworth Sleepiness Scale, and the Anxiety and Depression questionnaires.

**Results:**

Starting from the initial item data-bank, a 17-item questionnaire, the Maugeri Sleep Quality and Distress Inventory (MaSQuDI–17), was produced. Parallel Analysis on the MaSQuDI–17 confirmed the presence of a single dimension; exploratory factor analysis showed salient loading for each item, explaining 58.7% of total variance. Item-remainder correlation ranged from 0.72 to 0.39 and Cronbach alpha was 0.896. Rasch analysis revealed satisfactory psychometric properties of the new scale: the rating structure performed according to expectations, model fit was good and no item dependencies emerged. The scale presented good convergent validity and scores significantly distinguished healthy subjects from OSAS or Insomnia or BSD (p < 0.001).

**Conclusions:**

MaSQuDI –17 shows good psychometric qualities, and can be used to assess the impact of sleep disorders such as Insomnia, OSAS, Central Hypersomnia and BSD on emotional stress.

## Introduction

In recent years, increasing attention has been focused on the construct of *distress*, described as the impact of prolonged stress on quality of life [1].*Psychological distress* can be defined as a state of emotional suffering characterized by symptoms of depression (e.g. sadness, hopelessness), anxiety (e.g. feeling tense, ruminating), hyperarousal and psychophysiological tension that may be expressed through somatic symptoms like insomnia, headaches, muscular pain, lack of energy and exhaustion [1,2]. Although *distress* is a construct that embraces three domains—psychological, behavioral and somatic symptoms [1]—it is usually assessed with standardized scales such as *Hospital Anxiety and Depression Scale* (HADS)[3] or *Beck Depression Inventory* (BDI)[4], which are self-report questionnaires focused on the associated aspects of anxiety and depression symptoms [1,5,6]. In addition to these, there are tools that measure *non-specific distress* [7–9] such as the *Kessler Psychological Distress Scale* [10,11] which is widely used to screen for mental disorders in the middle-aged general population. Finally, there is also the *Psychological Distress Manifestations Measure Scale* (PDMMS) designed to explore comorbidity among symptoms [12], but it is not a diagnostic tool as it was developed in a non-clinical population [8].

Many studies have demonstrated the bidirectional relationship between distress and unhealthy sleep defined as sleep with a duration less than 7 hours or longer than 8 hours [7,9,13,14]. Evidence indicates that emotional distress is associated with changes in sleep architecture, total sleep time, sleep quality, sleep efficiency, rapid eye movement sleep, sleep onset latency, and slow wave sleep [9,15]. Moreover, short (≤6 hours) and long sleep duration (≥9 hours) have been shown to have a relationship with chronic disease, cerebrovascular disease, diabetes and mental health [16–20]. In the sleep disease literature, there is wide agreement that impaired sleep may directly contribute to the development of severe psychological discomfort or psychiatric disorders [13,21,22] both in the young and adult population. Patients with sleep apnea disorders often report anxiety, depression, irritability, or insomnia symptoms. In severe cases, Continuous Positive Airway Pressure (CPAP) therapy may improve anxiety and other psychological symptoms [23–28].

Based on the evidence highlighting the relationship between emotional distress, unhealthy sleep, sleep disorders and the disease impact [7,9,13,17,29,28], we focused our attention on psychometric instruments used to investigate psychological discomfort in patients suffering from a specific sleep disorder. However, to the best of our knowledge, no psychometric tools measuring the impact of sleep impairment on emotional distress are available in the literature, particularly in patients with sleep disorders. In order to fill this gap, we aimed to develop and validate a new questionnaire, the Maugeri Sleep Quality and Distress Inventory (MaSQuDI–17), to measure and monitor sleep-related distress in patients with Insomnia, Obstructive Sleep Apnea Syndrome (OSAS), Central Hypersomnia and Behavioral Sleep Disorders (BSD), a macro-category that includes unusual nocturnal behaviors such as Rapid Eye Movement (REM) Behavior Disorders, Parasomnia, Periodic Limb Disorders, Restless Legs Syndrome, Nocturnal Eating Disorders and Sleep Related Eating Disorders (American Academy of Sleep Medicine (2014) The international classification of sleep disorders: diagnostic and coding manual. 3nd edition, American Academy of Sleep Medicine, Westchester, Illinois). Furthermore, we considered that a psychometric tool specifically validated in subjects with a sleep disorder could be more sensitive in detecting the correlation between distress and unhealthy sleep in the general clinical population. In particular, measuring such discomfort in patients with chronic disabling disease can be helpful in optimizing the rehabilitation pathway.

## Material and methods

### Subjects

The study population consisted of consecutive outpatients evaluated for sleep disorders in various Sleep Centers of Northern Italy (the ICS Maugeri Scientific Institutes of Pavia, Tradate and Veruno; San Raffaele Hospital, Milano; and the Institute of Neurology, Casimiro Mondino Foundation, Pavia) in the period 2013–2015. We excluded individuals diagnosed with a serious psychiatric disease, neurological disorders, comorbidities that interact with the sleep mechanism, and sleep disorders, and those unable to read and fill in a simple questionnaire. In the end, we enrolled a total study sample of 357 subjects (age range 14–80 years), divided into four clinical groups: 267 with OSAS (mean age 52.4±13.6 years), 55 with chronic Insomnia (mean age 46.9±16.9 years), 24 with BSD (including REM Behavior disorders, Parasomnia, Periodic Limb Disorders, Restless Legs Syndrome, Nocturnal Eating Disorders and Sleep Related Eating Disorders) (mean age 53.9±17.2 years), and 11 patients with Central Hypersomnia (Narcolepsy type 1 and 2) (mean age 41.5±20.1 years). We also recruited 100 voluntary subjects who never had a diagnosis of sleep matched for the main sociodemographic characteristics (mean age 41.2±15.6 years). The ethics committee of the Salvatore Maugeri Foundation (867 CEC– 07/01/2013) approved the study. Each subject gave written informed consent to the protocol; for subjects under 18 years, parents gave their written informed consent.

### Procedures and participants

#### Item selection

1. Items were identified from other questionnaires that might fit our purpose to investigate and monitor sleep-correlated distress in patients with Insomnia, OSAS, Central Hypersomnia and BSD. The selection of items was based on clinical knowledge. We selected items pertaining to sleep-related psychological symptoms of stress and somatic perceptions, in particular depressive mood, anxiety, panic-fear feeling, rumination, concentration or memory problems, weakness, nervousness, tachycardia, over-sweating, and abdominal ache. The item data-bank was created which included domains of normal daily routine, social interactions, emotional functioning, and symptoms. Given the exploratory approach of our study, we used a 10:1 subject-to-variable ratio, a widely used rule-of-thumb in EFA Analyses, thus limiting the maximum number of items of the new questionnaire to 36; these items were discussed by a group of sleep specialists and health psychologists to remove duplicate or ambiguous items.
2. We organized two focus groups. The first focus group was requested to use the item data-bank as a starting point to identify other aspects that they felt could be affected by their disorder. In the second group, participants were enquired to define each item as ‘‘appropriate”, ‘‘not appropriate” or ‘‘unclear”, with the aim of achieving a consensus for each item. If an item was described as ‘‘not appropriate” or ‘‘unclear”, participants were asked to explain their reasons.
3. At the end of this process, the expert panel selected 36 items as suitable for the questionnaire, each with 4 response levels (‘Never’, ‘Sometimes’, ‘Often’, ‘Always’).
4. These data underwent a refining process using Classical Test Theory (CTT) and Item Response Theory (IRT) under the supervision of a panel of sleep specialists and health psychologists, in order to remove less relevant or ambiguous items. The remaining items formed a questionnaire, the Maugeri Sleep Quality and Distress Inventory (MaSQuDI–17).

### Measurements

Data on the subjective sleep quality were collected from the Pittsburg Sleep Quality Index (PSQI), data on sleepiness from the Epworth Sleepiness Scale (ESS), and data on anxiety and depression were assessed using the A-D Schedule.

PSQI [30]: this questionnaire collects information on night-time and daytime complaints over the past month in patient samples. It has seven components: subjective sleep quality, sleep latency, sleep duration, habitual sleep efficiency, sleep disturbances, use of sleep medication, and daytime dysfunction.

A-D Schedule: this consists of the State-Trait Anxiety Inventory (STAI-X1) [31] and the Depression Questionnaire (DQ) [32,33]. The STAI-X1 has a Cronbach alpha value equal to .92, contains 20 items based on a 4-point Likert scale and asks the respondent how they feel “right now”. The total score ranges from 20 to 80. The DQ explores and quantifies the presence of depressive symptoms, and is a 24-item self-report measure of depressive symptoms developed in Italy. Originally constructed in reference to the Diagnostic and Statistical Manual of Mental Disorders (DSM)-III, the questionnaire still satisfies all of the DSM-V criteria for a Major Depressive Disturbance (depressed mood; loss of interest or pleasure; variations in appetite and weight; insomnia/hypersomnia; psychomotor agitation/slowing; fatigability; self-depreciation; poor concentration; recurrent thoughts of death) [34]. The Cronbach alpha value is .86. Each item provides a statement (e.g. “I often feel like crying”) to which the response is Yes or No. The score ranges from zero to 24. Like STAI-X1, the instructions specify that the answers are to be made “thinking about how you feel at this moment”.

ESS [35]: this is an 8-item questionnaire that asks participants to rate their general tendency to doze off during the day, using a 4-point scale ranging from *would never doze* to *high chance of dozing*. We administered the ESS to obtain a measure of self-reported sleepiness.

Finally, we added two multiple-choice questions to evaluate the perceived restfulness on waking (“*Usually when you wake up in the morning you feel…*”) and sleep duration (*How many hours do you sleep at night*?”).

### Statistical analysis

#### Development phase

Classical Test Theory. In order to evaluate consistency, item-remainder correlation was used to examine the correlations between each item and the sum of the remaining items, omitting that item from the total. Spearman’s coefficient ρ greater than 0.40 was considered as the minimum value for satisfactory correlation [36]. Dimensionality was investigated using Factor Analysis (FACTOR software) [37]: an estimate of the number of factors in the responses was obtained with Parallel Analysis (PA) [38] methods, then an Exploratory Factor Analysis [39] for ordinal data was carried out to study the contribution of each item to the factors previously identified. The aim of this step was to detect possible additional dimensions to the one we were interested in (*sleep-correlated distress*) and flag for further study the pertaining items.

#### Item response theory

A more in-depth examination of the matrix of item responses was performed using Rasch Analysis (Winsteps software analysis program, version 3.69.1.96): using a rating scale model, a sample size of 100 subjects allows to estimate the item calibrations within ±½ logit with a 95% confidence [40].

The study sample size (n = 357) exceeded the 300 subjects suggested in the presence of a small number of factors and moderate-to-high factor loadings [41] and it was sufficient for Rasch Analysis to obtain stable calibration of items within ± 0.5 logits with 99% confidence [40].

The following steps were followed, in an iterative process, to successively refine the item set:

1. Rating scale diagnostic to investigate whether the rating scale was being used in the expected manner. We evaluated the response categories according to the criteria suggested by Linacre [42]: 1) at least 10 observations per category; 2) monotonic increase in both average measures across rating-scale categories: the average measure for a category is the average ability of the people who respond in that category; 3) threshold differences greater than 0.81 and less than 5 logits [43]. Thresholds (sometimes also called step calibrations) are the points at which the probability of a response in 1 or other of 2 adjacent categories is equally likely; i.e. thresholds represent the transition from one category to the next; 4) category outfit mean square values less than 2.
2. Validity assessment. We evaluated the goodness of fit of the real data to the modelled data, to test if there were items that did not fit the model expectations. We considered MnSq >0.7 and <1.3 as an indicator of acceptable fit [44]. Items outside this range were considered underfitting (MnSq >1.3, suggesting presence of unexpectedly high variability), or overfitting (MnSq <0.7, indicating a too predictable pattern).
3. Reliability was evaluated in terms of separation defined as the ratio of the person (or item) "true" standard deviation to the error standard deviation [45,46]. Item separation is used to verify the item hierarchy and reflects the number of “strata” of measures that are statistically discernible. A separation of 2.0 is considered good and sufficient to allow stratification into three groups [46]. A related index is the reliability of these separation indexes which provides the degree of confidence that can be placed in the reproducibility of these estimates; the value of the coefficient varies from 0 to 1 (values >0.80 are considered as good, and >0.90 excellent) [45].
4. Principal component analysis (PCA) on the standardized residuals was used to investigate:
  - The absence of subdimensions, as an independent confirmation of the dimensionality of the scale. In this case “unidimensionality” assumes that–after the removal of the trait that the scale is intended to measure (the “Rasch factor”), the residuals will be uncorrelated and normally distributed (i.e. there will be no principal components). The following criteria were used to determine whether additional factors were likely to be present in the residuals: at least 50% of the variance explained by the Rasch factor, eigenvalue of the first contrast smaller than 3, and variance explained by each contrast smaller than 5%.
  - The local independence of items. High correlation (>0.30) of residuals for two items indicates that they may not be locally independent or there is a subsidiary dimension in the measurement which is not accounted for by the main Rasch dimension [47].

Based on the results of the analyses and expert opinion, changes were made; the remaining items again underwent analysis, until the expert panel considered it had attained a satisfactory solution. A final Classical Test Theory run was performed to consolidate the psychometric properties of the new questionnaire.

Validation phase. The convergent validity of the new scale with the PSQI, ESS and A-D Schedule was investigated by regression analysis. The concurrent validity was evaluated against “Perceived restfulness at wake up” and “Sleep Duration”. The difference in scoring on the new scale was tested between normal and pathological subjects using a t-test for unpaired data. The capability of the scale to detect differences in sleep-correlated distress between various pathological conditions (OSA, INS, BSD) compared to healthy subjects was finally tested with a t-test for unpaired data.

## Results

**Fig 1:**
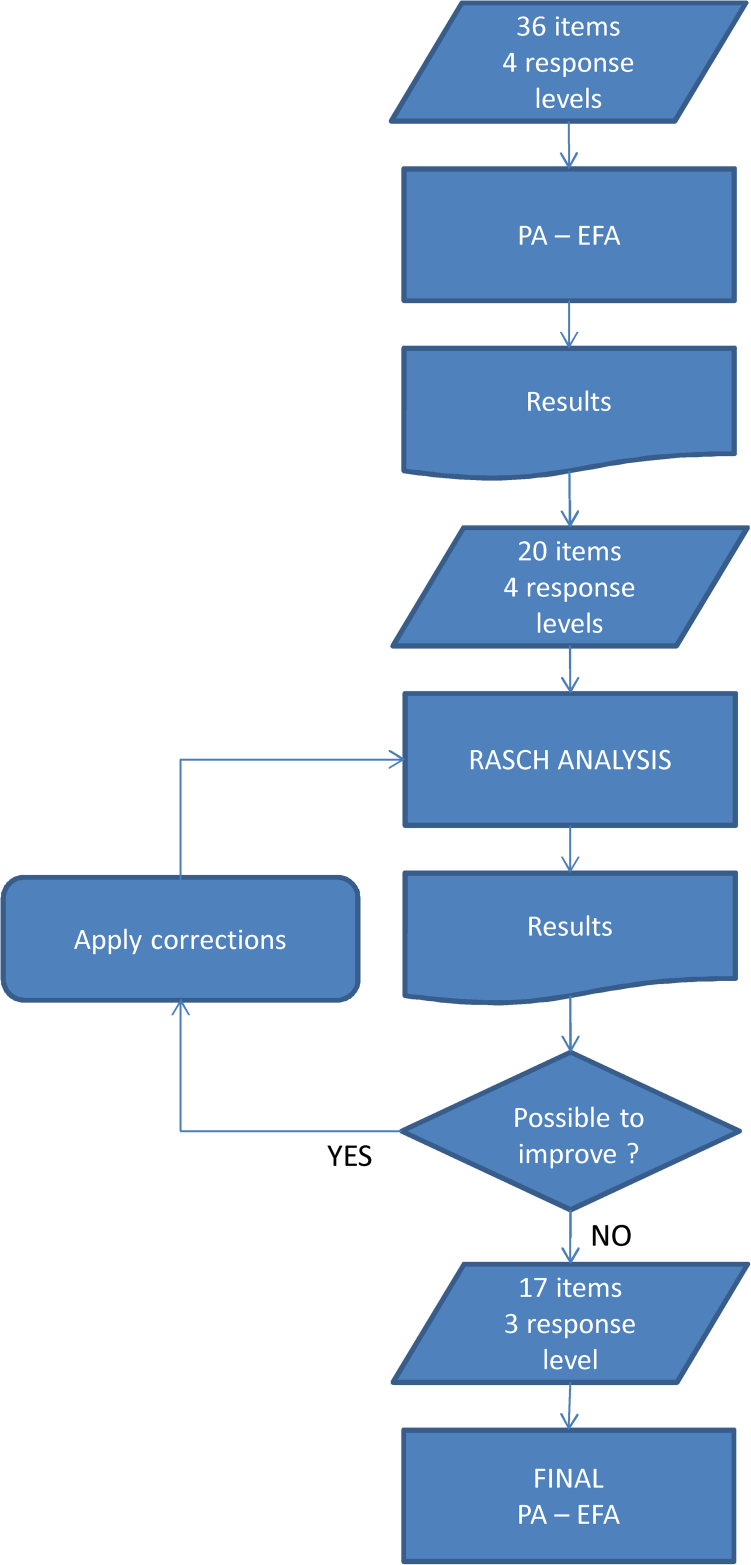
Analysis flow chart of the procedure for development and validation of the MaSQuDI-17.

The final item bank, composed of 17 items, was administered to a sample of 357 subjects and 100 subjects without sleep disorders. Table 1 summarizes the demographic and clinical characteristics of the study sample. A flow chart of the analytical steps performed in the development and validation phases is presented in Fig 1.

**Table 1:** Main characteristics of the sample enrolled.

		OSAS	INS	BSD	C HYPER	NORM
	N° (% of total sample)	267 (58.4)	55 (12%)	24 (5.2)	11 (2.4)	100 (21.9)
	age± sd	52.4±13.6	46.9±16.9	53.9±17.2	41.5±20.1	41.2±15.6
	BMI (Kg/m^2^) ± sd	31.3±8	25.4±4.1	25.3±3.6	25.9±4.4	23.5±4.3
	Male sex N (%)	176 (65.9)	25 (45.4)	15 (62.5)	15 (72.7)	45 (45)
**Sleep Duration (%)**	less than 6 h	111 (41.6)	36 (65.4)	16 (66.7)	3 (27.3)	16 (16)
	6–8 h	131 (49.1)	10 (18.2)	8 (33.3)	5 (45.4)	74 (74)
	8–10 h	13 (4.9)	1 (1.8))	0 (0)	3 (27.3)	9 (9)
	do not know	10 (3.7)	6 (10.9)	0 (0)	0 (0)	1 (1)
	NA	2 (0.7)	2 (3.6)	0(0)	0 (0)	0 (0)
**Sleep Quality (%)**	Rested	78 (29.2)	3 (5.4)	3 (12.5)	5 (45.4)	55 (55)
	Just rested	125 (46.8)	24 (43.6)	9 (37.5)	4 (36.7)	33 (33)
	Tired	60 (22.5)	27 (49.1)	12 (50)	2 (18.9)	11 (11)
	NA	4 (1.5)	1 (1.8)	0 (0)	0 (0)	1 (0.9)

Legend: OSAS = Obstructive Sleep Apnea Syndrome; INS = Insomnia; BSD = Behavioral Sleep disorders; NORM = Normal; C Hyper = Central Hypersomnia; BMI = body mass index; NA = not applicable.

### Development phase

#### Classical test theory

**Fig 2:**
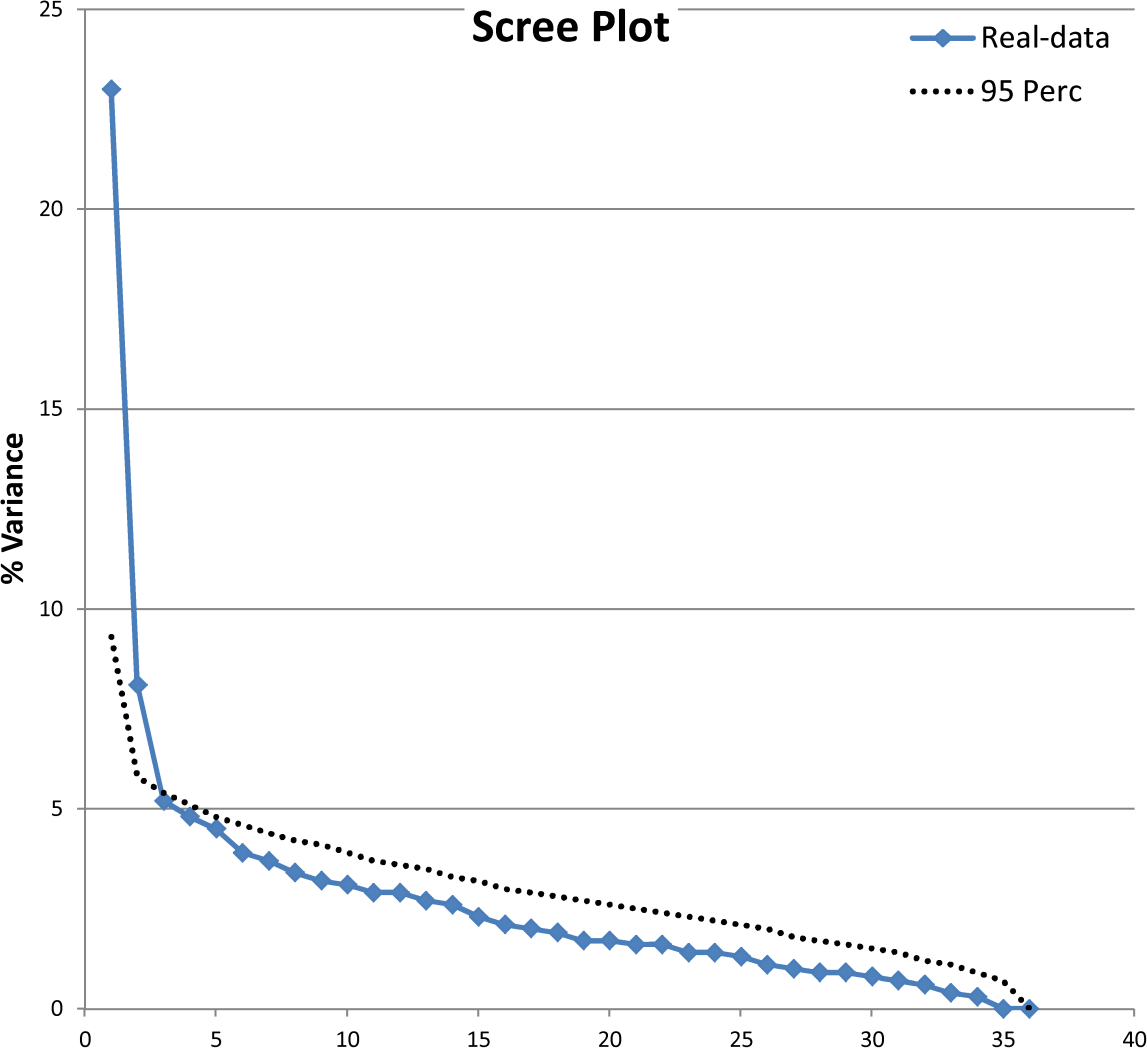
Parallel analysis—development phase–initial item sets.

Item-remainder correlation showed ρ ranging from 0.03 (item 10) to 0.72 (item 23): 12 items were under the 0.4 threshold. Parallel Analysis suggested the presence of 2 factors (Fig 2). The relationship between items and these factors is presented in Table 2 in the column ‘36 items’ (loading factors under 0.3 omitted). Orthogonal or oblique rotation did not alter the item distribution between the factors. The two factors appeared orthogonal since an oblique rotation did not alter the item distribution; the main factor was judged as pertaining to the latent trait of interest with the second more dependent on a wide variety of symptoms connected to different sleep disorders. The items with low item-remainder correlation were weakly associated to any factor. The total amount of variance explained by the two dimensions was 43.6%.

**Table 2:** Factor analysis: Varimax and Promax rotations in the 36-item questionnaire, 2 factors as per Parallel Analysis. Single factor analysis for the reduced item set (17 items, Final version). Factor loadings < .3 omitted.

	36 items				17 Items
	VARIMAX Rotation		PROMAX Rotation		-
Item	*Factor 1*	*Factor 2*	*Factor 1*	*Factor 2*	*Factor 1*
1	0.401		0.371		
2	0.347		0.325		
3	0.652		0.619		0.671
4		0.314		0.305	
5	0.639		0.594		0.664
6	0.614		0.615		0.736
7	0.818		0.789		0.830
8	0.68		0.638		0.671
9	0.587		0.594		0.729
10					
11		0.53		0.528	
12	0.554		0.574		0.712
13		0.359		0.356	
14		0.481		0.47	
15		0.566		0.545	
16	0.648		0.642		0.692
17	0.671		0.659		0.795
18		0.636		0.632	
19	0.349		0.373		
20			0.304		0.554
21		0.339		0.333	
22		0.616		0.628	
23	0.767		0.762		0.824
24	0.413		0.412		0.559
25		0.746		0.706	
26	0.739		0.73		
27	0.432		0.433		
28	0.467	0.312	0.521	0.383	0.657
29					
30	0.525		0.533		0.649
31			0.308		0.552
32	0.609		0.59		0.678
33		0.767		0.742	
34	0.322		0.352		0.500
35					
36	0.382		0.387		

#### Rasch analysis

**Fig 3:**
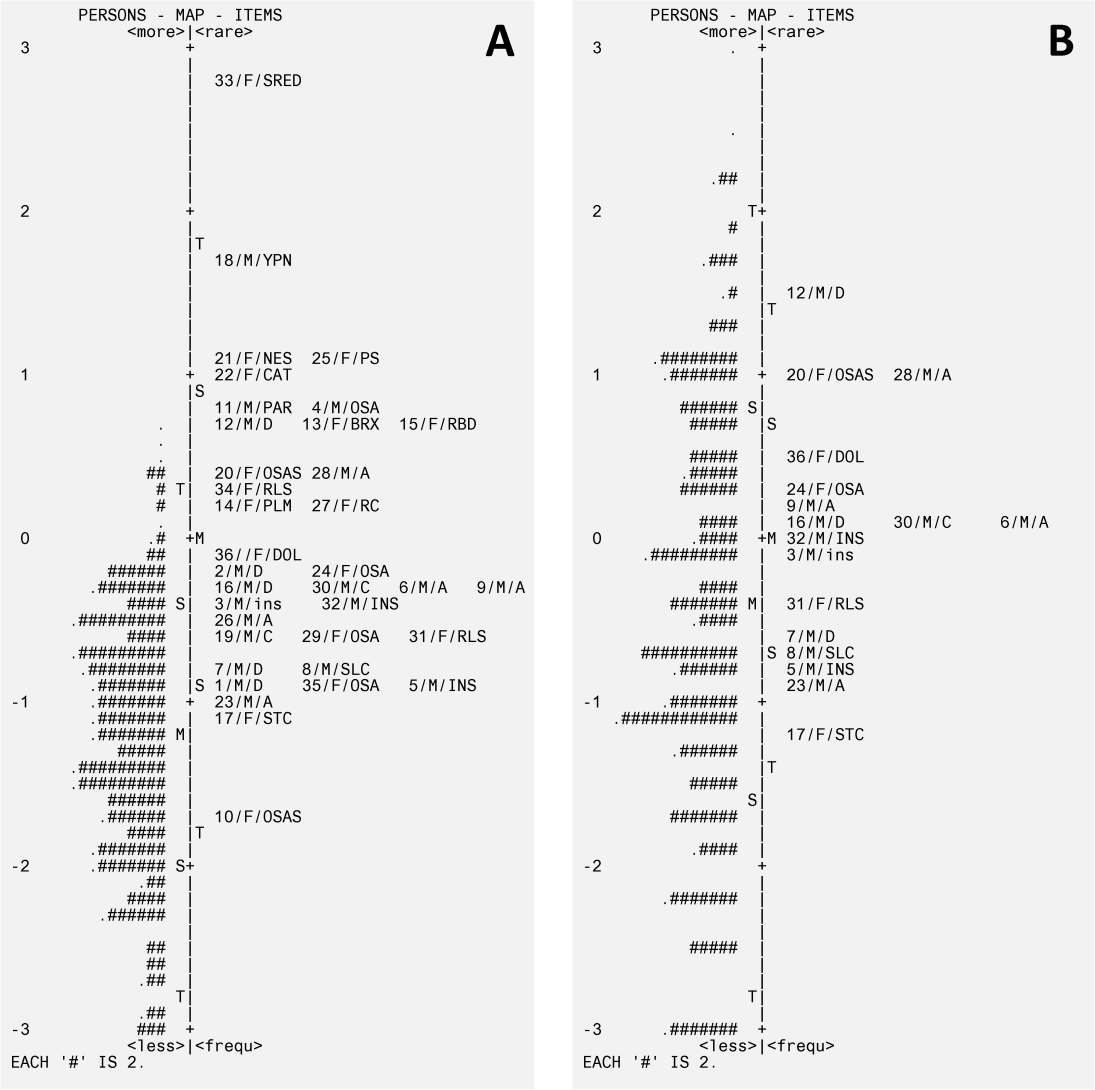
Rasch analysis—persons item map. (A) Starting version of 36 items. (B) Final item set, after recoding, 17 items.

**Fig 4:**
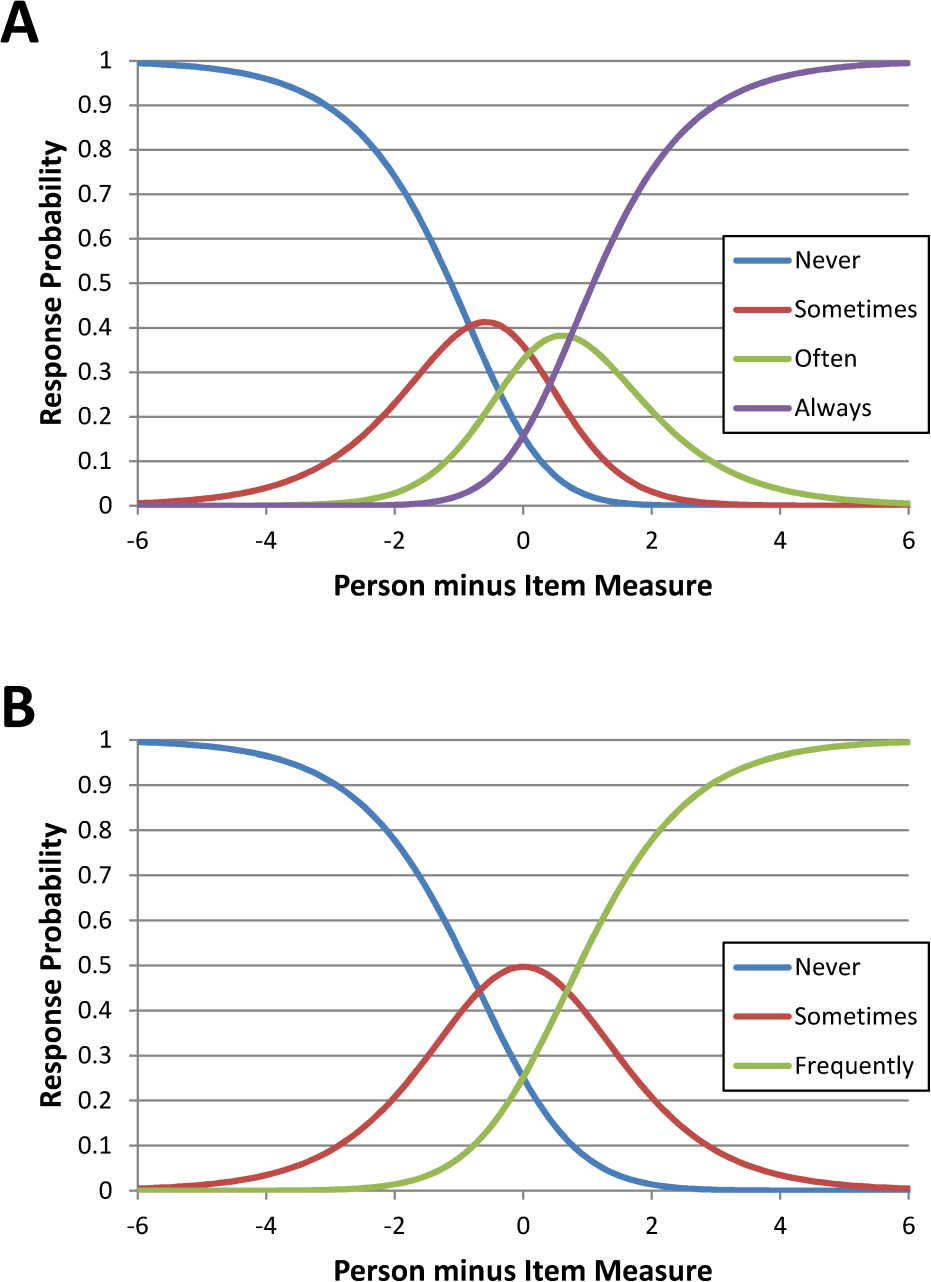
Category probability curves—development phase. (A) Development phase for the 4-point Likert rating scale. (B) Development phase–for the 3-point Likert choice for the final 17-item set after recoding.

Rasch Analysis identified a number of issues in the main collection of items. We examined the map of persons and items to compare the range and position of the item measure distribution to the range and position of the person measure distribution. Fig 3A shows the Map of Persons and Items in the 36- and 17-item versions (Fig 3B). The rating structure did not perform in a satisfactory way, with an under-utilization of categories 3 and 4 ‘Often’ and ‘Always’; this fact also produced non-monotonicity of the response thresholds in three items (11, 22, 25) (Fig 4A). A few items did not fit the Rasch model, see Table 3, and there were item dependencies (between items 1 and 5, 15 and 25, 3 and 26, 34 and 36). Mean person measure was -1.23 (max = 0.7, min = -3.61, separation = 2.69, reliability = 0.88).

**Table 3:** Rasch analysis–start and final item set FIT tables.

	Start				Final			
Item	MEASURE	S.E.	INFIT MNSQ	OUTFIT MNSQ	MEASURE	S.E.	INFIT MNSQ	OUTFIT MNSQ
1	-0.87	0.06	1.17	1.14				
2	-0.21	0.07	1.31	1.27				
3	-0.42	0.07	0.93	0.90	-0.06	0.09	1.04	1.06
4	0.85	0.10	1.47	1.38				
5	-0.93	0.06	0.96	0.92	-0.77	0.09	1.09	1.17
6	-0.30	0.07	0.86	0.80	0.06	0.09	0.93	0.89
7	-0.79	0.07	0.58	0.58	-0.70	0.10	0.74	0.73
8	-0.81	0.06	0.80	0.79	-0.71	0.09	1.04	1.08
9	-0.27	0.07	0.88	0.87	0.14	0.09	0.92	0.97
10	-1.70	0.07	1.89	2.12				
11	0.84	0.10	1.20	1.26				
12	0.70	0.09	1.06	0.84	1.31	0.10	0.92	0.81
13	0.65	0.09	1.49	1.55				
14	0.15	0.08	1.36	1.26				
15	0.72	0.09	1.12	1.11				
16	-0.28	0.07	0.77	0.75	0.13	0.09	0.98	0.94
17	-1.10	0.06	0.56	0.56	-1.33	0.10	0.81	0.80
18	1.73	0.14	1.16	1.00				
19	-0.65	0.07	0.79	0.81				
20	0.37	0.08	1.04	0.97	1.03	0.10	1.16	1.25
21	1.12	0.11	1.53	1.64				
22	0.98	0.10	1.25	1.14				
23	-0.96	0.06	0.48	0.49	-1.08	0.10	0.76	0.74
24	-0.16	0.07	1.02	0.98	0.32	0.09	1.20	1.24
25	1.07	0.11	1.20	1.13				
26	-0.53	0.07	0.59	0.58				
27	0.20	0.08	1.05	0.94				
28	0.38	0.08	0.88	0.81	1.01	0.10	0.99	1.04
29	-0.57	0.07	1.29	1.41				
30	-0.31	0.07	0.82	0.80	0.09	0.09	1.02	1.02
31	-0.63	0.07	1.06	1.11	-0.38	0.09	1.24	1.22
32	-0.40	0.07	1.07	0.99	0.00	0.09	1.09	1.08
33	2.80	0.24	1.81	1.08				
34	0.27	0.08	1.29	1.15	0.93	0.10	1.22	1.16
35	-0.85	0.06	1.07	1.09				
36	-0.07	0.07	1.09	1.02				

Following expert opinion, the following changes were cumulatively applied: 1) items loading on the second factor according to exploratory factorial analysis (FA) were excluded; 2) the rating scale was reduced to 3 levels (“Never”, “Sometimes”, “Frequently”, Fig 4B); 3) RA misfitting items were excluded; 4) for each item pair showing dependencies, the most clinically relevant item was retained and the other excluded.

**Fig 5:**
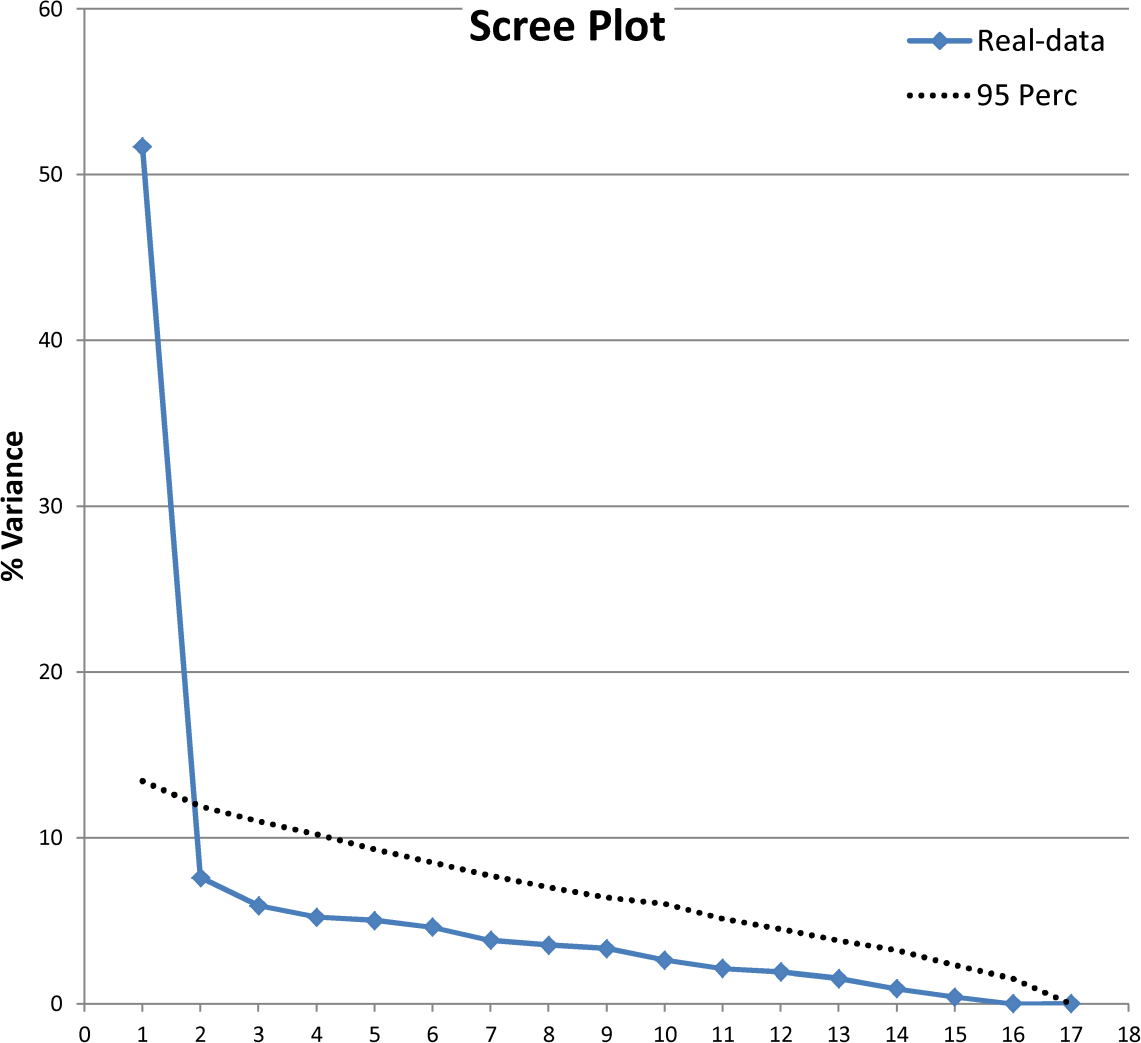
Parallel analysis—development phase—final item sets.

The final item set was composed of 17 items: never = 1; sometimes = 2 and frequently = 3. The new rating structure performed according to expectations. All items fitted the model, with slight overfitting in items 7 and 23 (‘items too predictable’). No item pair showed any dependence and mean person measure was -0.37 (max = 3.73, min = -3.71, separation = 2.45, reliability = 0.86). Parallel analysis applied to the 17-item recoded data set confirmed the presence of a single dimension (Fig 5); exploratory factor analysis showed salient loading for each item (Table 2 –‘17 items’ column), with 58.7% of total variance explained. Item-remainder correlation ranged from 0.72 (item 23) to 0.39 (item 20). Cronbach alpha was 0.896.

#### Validation phase

**Fig 6:**
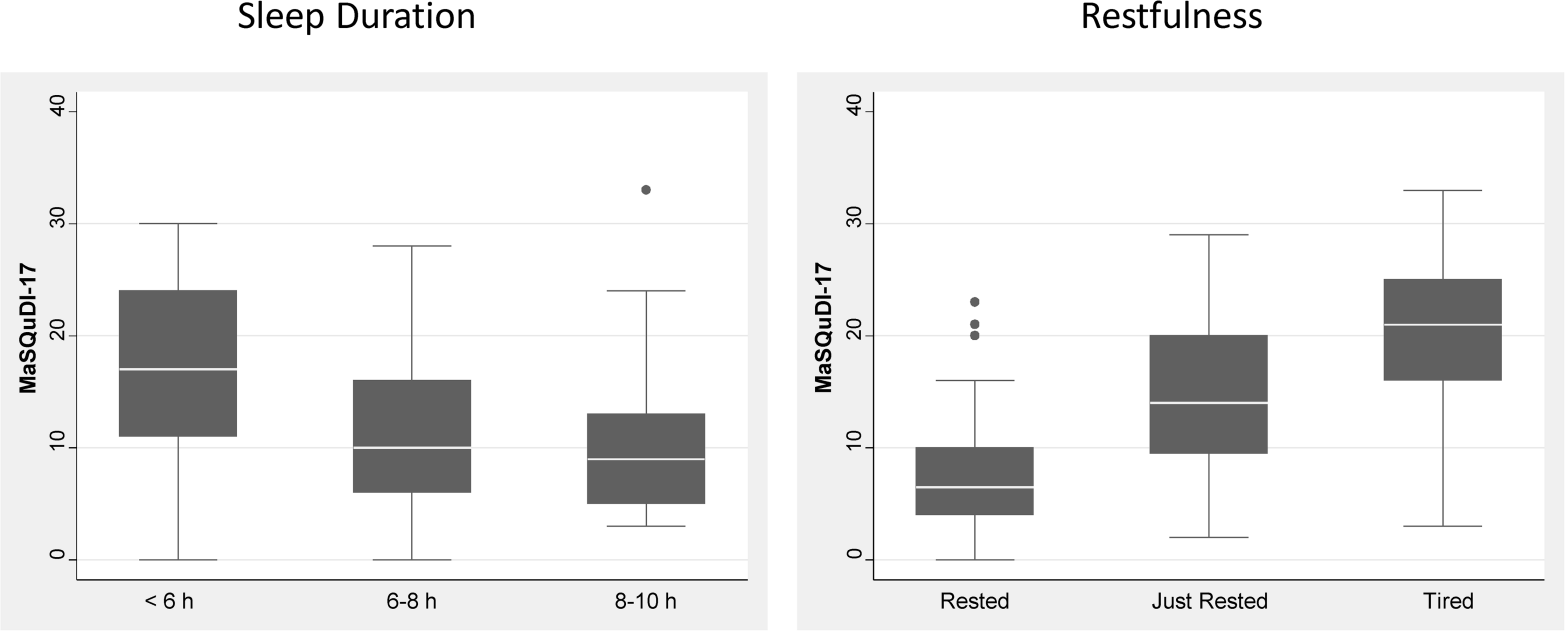
Concurrent validity of the MaSQuDI–17scores according to subjective sleep duration and perceived restfulness at wake up.

**Fig 7:**
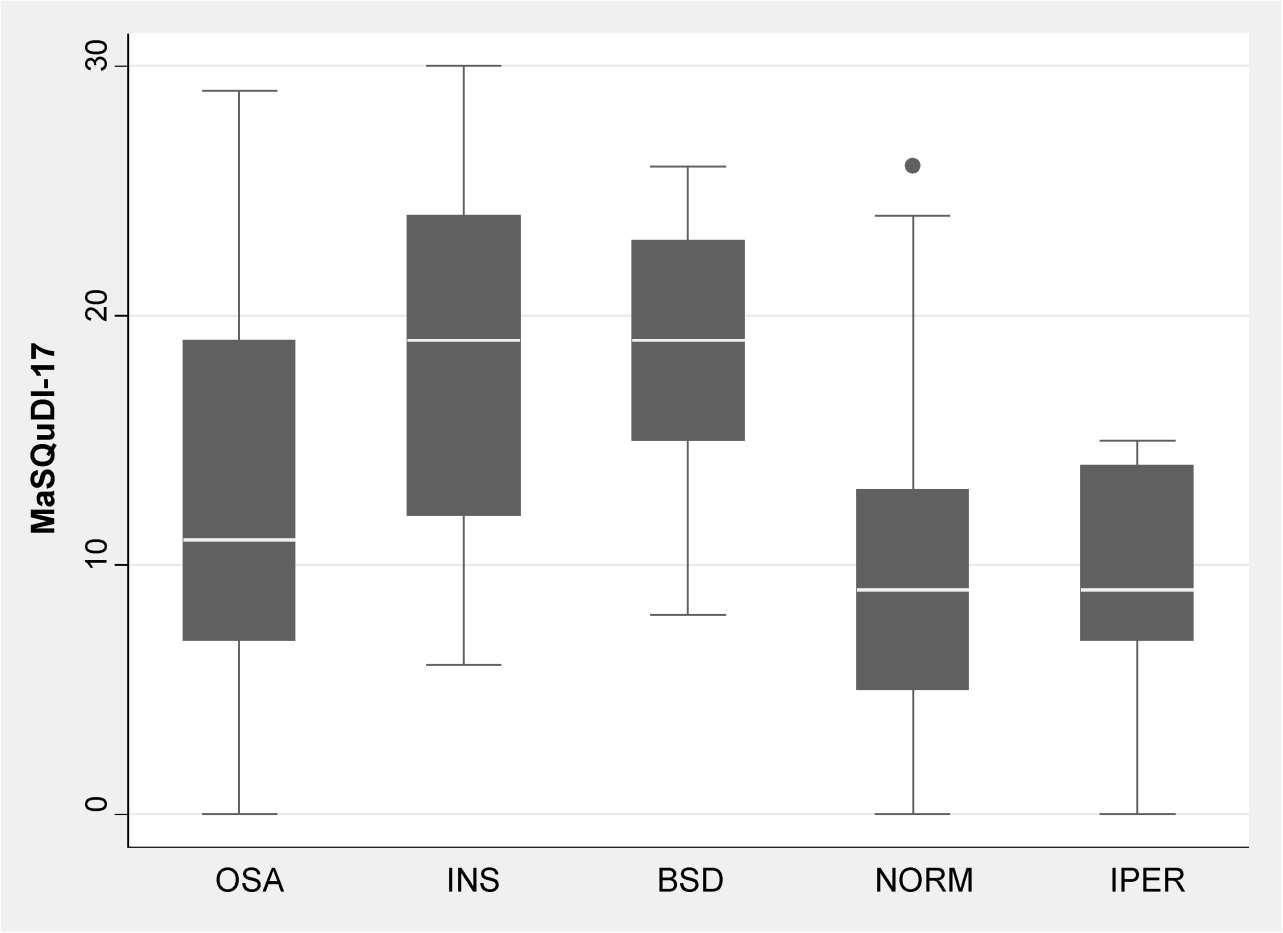
Box plot showing the distribution of mean scores and percentile for subjects suffering from Obstructive Sleep Apnea Syndrome, Insomnia, Behavioral Sleep Disorders and Central Hypersomnia.

The score computed using the new 17-item scale presented good convergent validity: r^2^ = 0.5 with the PSQI (p < 0.001), r^2^ = 0.15 with ESS (p < 0.001), r^2^ = 0.39 with anxiety as measured by A-D schedule (p < 0.001), and r^2^ = 0.52 with depression as measured by A-D (p < 0.001). Concurrent validity with “Perceived restfulness at wake up” and “Sleep Duration” is graphically presented in Fig 6. The difference in mean score between healthy group and patients (9.31 and 14.15 respectively) was statistically significant (p < 0.001), and differences were consistent with the type of pathological condition present (normal subject vs. OSAS or INS or BSD group of subjects p < 0.001), but not with the group affected by Central Hypersomnia which comprised only 11 subjects (Fig 7).

## Discussion

The aim of this study was to develop a scale for measuring patients’ perceptions of the impact of sleep impairment on emotional distress in patients with various sleep disorders and evaluate the psychometric properties of the resulting questionnaire (MaSQuDI–17) using a mix of classical test theory and IRT methods to provide reliability and validity estimates. The qualitative phase of the study identified the items that are needed for breadth, range and precision of measurement. Then, we focused our attention on analysis of dimensionality, rating scale diagnostics and identification of those items most useful for measuring the intended construct (model fit).

Rating scale diagnostics provided evidence that respondents were unable to discern appreciably the response levels proposed by the preliminary 36-item questionnaire. The collapsing procedure produced a simpler 3-level rating scale (“Never”, “Sometimes” and “Frequently”). This procedure improved the measurement qualities of the scale (increasing its reliability indexes), minimized irrelevant construct variance and ensured that each rating category represents a clearly distinct level of agreement. Rasch validation of the MaSQuDI–17 confirmed the scale’s unidimensional nature at both PA and the appropriateness of its simplified rating categories. Item fit to the Rasch model, on the other hand, confirmed the final 17-item set and the explained variance of the unidimensionality was satisfactory. The results of PCA on standardized residuals showed that the latent trait measured by the questionnaire is sufficiently unidimensional. Regarding reliability indexes, the high values of item separation reliability indicates good replicability of item placement in other samples and the ability to define a distinct hierarchy of items. In the same way, the person separation index indicates the ability to detect three strata of patients along the construct “impact of sleep disorders on emotional distress”.

In the end, we obtained a 17-item questionnaire based on a 3-point Likert scale (1 = never, 2 = sometimes, 3 = frequently) with a total score ranging from 17 to 51. Higher scores at MaSQuDI–17 indicate the presence of greater sleep-related distress.

The high correlation of the questionnaire with the questionnaires measuring anxiety and depression confirms the construct validity of MaSQuDI–17. In fact, higher scores on the questionnaire were associated to a higher level of anxiety and depression on the A-D Schedule. These results are in line with the sleep disease literature, in which there is a wide consensus that impaired sleep may directly contribute to the development of severe psychological discomfort or psychiatric disorders [13,21,22] both in the young and adult population. For instance, insomnia has been shown to increase the likelihood of developing subsequent depression [48,49]. Moreover it is known that patients with respiratory sleep disorders suffer from insomnia, irritability, depression or anxiety disorders, affecting negatively their quality of life [50–52]. Recently, in a sample of severe OSA, Lee et al. [53] found that the strongest predictor of depression symptoms, measured with the BDI, was sleep quality.

Finally, internal consistency of the MaSQuDI–17 showed an alpha Cronbach value adequate for its clinical application [54], and the convergent validity was proven by the good correlations with PSQI and ESS, showing the questionnaire to be an adequate instrument to investigate the subjective quality of sleep. Furthermore, the concurrent validity between MaSQuDI–17 and the two questions about the “perceived restfulness at wake up” and “sleep duration” confirms previous findings that demonstrated the bidirectional relationship between distress and unhealthy sleep [7,9,13,15]. Indeed, our study subjects suffering from a sleep disorder reported a worse restfulness on waking as well as a shorter sleep duration and scored higher on the MaSQuDI–17 than the “normal” sample of subjects. These results endorse the association of distress with any sleep disorder, and highlight the necessity to measure the dimension, which has strong implications on patients’ quality of life.

Seixas et al. [9] suggested that emotional distress could be considered as a significant predictor of unhealthy sleep, independently of the presence of other health risk factors or different chronic diseases. Their study highlighted the importance of assessing emotional distress among individuals experiencing unhealthy sleep. Cunningham et al. [7] reached a similar conclusion with a population-based data analysis study. They emphasized that not only is there a higher likelihood of having unhealthy sleep together with Serious Psychological Distress (SPD), but even that any level of psychological distress is associated with unhealthy sleep and hence requires adequate management. Gianfagna et al. [55] suggested that a short questionnaire assessing levels of sleep disturbances and sleep duration should be routinely adopted in cerebrovascular disease prevention programs to identify people at increased risk. However, few studies have applied specific tools to evaluate sleep-related distress, and the MaSQuDI–17 may fill this gap.

The main limitation of this study is the relative small sample size which, although sufficient for the statistical analysis of the questionnaire, could have been larger in order to better balance the different sleep disorders represented. Furthermore, in our sample we considered only four major classes of sleep disorders, one of which (BSD) includes various different disorders: future research efforts could further differentiate the heterogeneity of these disorders in order to obtain a more sensitive tool.

## Conclusion

Based on the published literature, the importance of assessing and managing psychological distress in different health conditions is clear. We have demonstrated the MaSQuDI–17 to be a robust and comprehensive measure of psychological distress related to sleep disorders. Considering the bidirectional relationship between distress and unhealthy sleep, our questionnaire could be used to investigate this psychological construct also in other clinical populations. It has been shown that sleep duration and sleep quality are correlated to chronic disease, cerebrovascular disease, diabetes and mental health [16–20]. We think that our questionnaire could be used for an early detection of the impact of distress related to sleep disturbance. Sleep disorder is more often associated to psychological symptoms, although anxiety and depressive symptoms worsen sleep quality and sleep disorder development. In particular, patients with sleep-related breathing disorders and insomnia report more of these symptoms, and few instruments are available to evaluate these aspects. Future research could be oriented to study sleep-related distress in other medical conditions such as chronic diseases. The MaSQuDI–17 may be useful to identify different levels of sleep-related distress in patients suffering from a sleep disorder during their treatment. In fact, it appears to be a sensitive tool to monitor sleep-related distress variation in relation to the efficacy of treatments. Further studies are necessary to determine its accuracy to evaluate pre- and post-treatment.

## Supporting information

S1 File This is the S1 file MaSQuDI-17.pdf. This is the complete MaSQuDI-17 test.
(PDF)

S2 File This is the S2 file database.xlsx. This is the complete MaSQuDI-17 database.
(XLSX)
