# Supplementary material for: Development and validation of the Maugeri Sleep Quality and Distress Inventory (MaSQuDI-17)
Source: PLoS One. 2017 Jul 10;12(7):e0180743. doi: 10.1371/journal.pone.0180743 (PMC5507265; doi:10.1371/journal.pone.0180743)
Supplement: S1 File — This is the complete MaSQuDI-17 test. (PDF) [file pone.0180743.s001.pdf]

Istituti Clinici Scientifici Maugeri

Maugeri Sleep Quality and Distress Inventory (MaSQuDI-17)

	Never	Sometimes	Frequently
1. I have unpleasant thoughts, which I can't get rid of	☐	☐	☐
2. I wake up several times during the night and have difficulty getting back to sleep	☐	☐	☐
3. I can be overcome all of a sudden by a physical ailment (e.g. heart beating quickly, sweating, tremors, stomach ache, shortness of breath)	☐	☐	☐
4. I feel down in the dumps	☐	☐	☐
5. I feel stressed in relation to work and/or economic-family problems	☐	☐	☐
6. I feel apprehensive as if something was about to happen	☐	☐	☐
7. I cry for no reason at all	☐	☐	☐
8. I don't feel like doing the things that I used to like doing	☐	☐	☐
9. During the day I can feel physically worn out, even when I haven't done any strenuous activity	☐	☐	☐
10. I wake up all of a sudden feeling I'm unable to breathe or overcome by a sense of panic	☐	☐	☐
11. I feel tense and irritable	☐	☐	☐
12. I wake up with a headache	☐	☐	☐
13. I am unable to do an activity or task because I'm too nervous and tense	☐	☐	☐
14. I have difficulty concentrating during the daytime	☐	☐	☐
15. I have a strong need to move my legs especially when I have to remain still for a long time	☐	☐	☐
16. At night, I have the sensation that I can't stop my thoughts flowing and that my mind is racing.	☐	☐	☐
17. I have difficulty falling asleep due to an intense irritation in the legs	☐	☐	☐
